# 1,1,2,2-Tetra­kis(diisopropyl­amino)diphosphane

**DOI:** 10.1107/S1600536809032802

**Published:** 2009-08-22

**Authors:** Rafał Grubba, Łukasz Ponikiewski, Jarosław Chojnacki, Jerzy Pikies

**Affiliations:** a Chemical Faculty, Gdansk University of Technology, G.Narutowicza 11/12, Gdansk PL-80233, Poland

## Abstract

In the title compound, C_24_H_56_N_4_P_2_, the distance between the P atoms [2.2988 (8) and 2.3013 (13) Å in the major and minor occupancy components, respectively] is one of the longest reported for uncoordinated diphosphanes. The whole mol­ecule is disordered over two positions with site-occupation factors of 0.6447 (8) and 0.3553 (8). The structure adopts the synperiplanar conformation in the solid state [N—P—P—N torsion angle = 14.7 (5)°].

## Related literature

For reactions of diphosphanes with transition metal chlorides, see: Pikies *et al.* (2004 ▶). For related structures, see: Becker *et al.* (1999 ▶); Bezombes *et al.* (2004 ▶); Hinchley *et al.* (2001 ▶, 2004 ▶); Mundt *et al.* (1988 ▶); Bender *et al.* (1994 ▶).
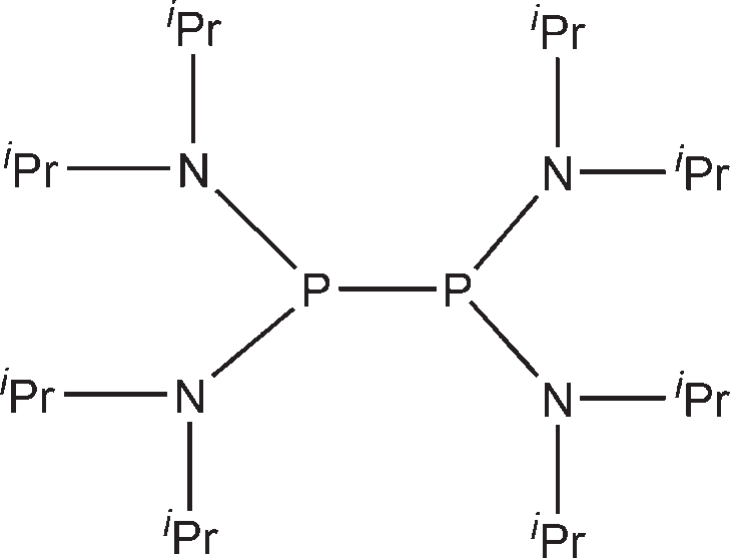


## Experimental

### 

#### Crystal data

C_24_H_56_N_4_P_2_
*M* *_r_* = 462.67
Monoclinic, 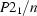

*a* = 11.601 (2) Å
*b* = 14.493 (3) Å
*c* = 17.280 (4) Å
β = 97.22 (3)°
*V* = 2882.2 (10) Å^3^
*Z* = 4
Mo *K*α radiation
μ = 0.17 mm^−1^
*T* = 150 K
0.38 × 0.23 × 0.21 mm

#### Data collection

Stoe IPDS 2 diffractometer
Absorption correction: none
20113 measured reflections
5601 independent reflections
4529 reflections with *I* > 2σ(*I*)
*R*_int_ = 0.032

#### Refinement

*R*[*F* ^2^ > 2σ(*F* ^2^)] = 0.038
*wR*(*F* ^2^) = 0.105
*S* = 1.03
5601 reflections
518 parameters
6 restraints
H-atom parameters constrained
Δρ_max_ = 0.30 e Å^−3^
Δρ_min_ = −0.17 e Å^−3^

### 

Data collection: *X-AREA* (Stoe & Cie, 1997 ▶); cell refinement: *X-AREA*; data reduction: *X-RED* (Stoe & Cie, 1997 ▶); program(s) used to solve structure: *SHELXS97* (Sheldrick, 2008 ▶); program(s) used to refine structure: *SHELXL97* (Sheldrick, 2008 ▶); molecular graphics: *ORTEP-3* (Farrugia, 1997 ▶); software used to prepare material for publication: *WinGX32* (Farrugia, 1999 ▶).

## Supplementary Material

Crystal structure: contains datablocks I, global. DOI: 10.1107/S1600536809032802/pv2196sup1.cif

Structure factors: contains datablocks I. DOI: 10.1107/S1600536809032802/pv2196Isup2.hkl

Additional supplementary materials:  crystallographic information; 3D view; checkCIF report

**Fig. 2.**
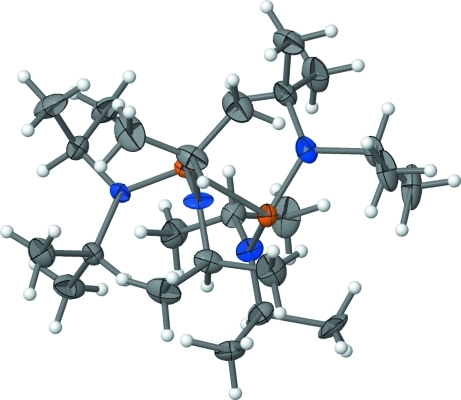
Jmol enhanced figure. Disorder in (I).

Enhanced figure: interactive version of Fig. 2

## Figures and Tables

**Fig. 1.**
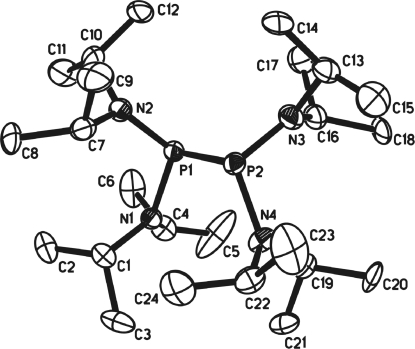
View of (I) (50% probability displacement ellipsoids), hydrogen atoms omitted. Only the more occupated part shown.

## supplementary crystallographic information

### Comment

In the course of our studies on phosphinophosphinidene ligands *R*_2_P–P we have investigated reactions of diphosphanes *R*_2_P—P(SiMe_3_)Li with transition metals chlorides (Pikies *et al.*, 2004). The title compound, (^i^Pr_2_N)_2_P–P(N^i^Pr_2_)_2_ (I), turned out to be the main product in the reaction of (^i^Pr_2_N)_2_P–P(SiMe_3_)Li with [Cp_2_ZrCl_2_] (mol ratio 2:1 in DME); (I) is formed as a result of cleavage of the P–P bond in the starting material, (^i^Pr_2_N)_2_P—P(SiMe_3_)Li. We observed the formation of diphosphanes (*R*_2_P–P*R*_2_) in every reaction of [Cp_2_ZrCl_2_] with *R*_2_P—P(SiMe_3_)Li in different yields. The formation of diphosphane in exceptionally preferred in the case of *R*=^i^Pr_2_N. The crystal structure of (I) is presented in this paper.

The whole molecule of the title compound was disordered over two positions with site occupation factors of 0.6447 (8) and 0.3553 (8) referred to as part A and B, respectively; the part A is presented in Fig. 1. The structure adopts the synperiplanar conformation in the solid state (N1—P1—P2—N4 torsion 14.7 (5)°). The most striking structural feature in (I) is P1–P2 bond length (2.2988 (8) Å in part A and 2.3013 (13) Å in  part B). This distance between phosphorous atoms is one of the longest reported for uncoordinated diphosphanes. There is only one report of diphoshane {P[CH(SiMe_3_)_2_]_2_}_2_ with a longer P–P bond  of 2.310 (7) Å (Hinchley *et al.*, 2001). Another diphosphane which contains amino grups [N(SiMe_3_)_2_](N^i^Pr_2_)P–P[N(SiMe_3_)_2_](N^i^Pr_2_) has slightly shorter P–P distance of 2.291 (4) Å (Bezombes *et al.*, 2004). These three diphosphanes *R*_2_P–P*R*_2_ posses *R* groups which have a form of –A*X*_2_, where A = N or CH and *X* are voluminous rests SiMe_3_ or ^i^Pr. The steric effects of bulky *R* groups like *^t^*Bu are not sufficient for such an elongation (Hinchley *et al.*, 2004) and for *^t^*Bu_2_P–P*^t^*Bu_2_ a P–P distance of 2.235 Å was reported. The presence of four nitrogen substituents in the molecule is not sufficient cause of the elongation either. For DMP(^i^Pr_2_N)P–P(N^i^Pr_2_)DMP (DMP = 2,6-dimethylopiperidine) the P–P distance of 2.259 (2) Å was reported (Bender *et al.*, 1994). The enhanced stabilities of related radicals P[CH(SiMe_3_)_2_] or P[N(SiMe_3_)_2_](N^i^Pr_2_) can be seen as a possible explanation of the elongation phenomena. These diphosphanes dissociate very easy into relatively stable radicals (Hinchley *et al.*, 2001).

To study repulsions of substituents around P-atom we analysed the average angle around P atoms. Its deviation from orthogonality (90°) can be a rough measure of the steric crowding. The average angle in (I) around P atoms is 105.98°. The widest ones, above 110°, are N—P—P angles for N1 and N4 (in disorder part A), perhaps because nitrogen atoms are in synperiplanar position. The N—P—P angles for N3 and N2 atoms are only 93.5 (6)° and 92.0 (4)°, respectively. For comparison, in the case of very small groups around P-atoms, i.e., for Me_2_P–PMe_2_ the average angle is 98.40° (close to orthogonality) (Mundt *et al.*, 1988) and for (F_3_C)_2_P–P(CF_3_)_2_ is only 96.67° (Becker *et al.*, 1999). For the most crowded diphosphanes, i.e., for *^t^*Bu_2_P–P*^t^*Bu_2_, this average angle is 109.99° (Hinchley *et al.*, 2004) and for [(Me_3_Si)_2_CH]_2_P–P[CH(SiMe_3_)_2_]_2_ is 105.23° (Hinchley *et al.*, 2001).

### Experimental

The work was carried out using the standard vacuum-nitrogen line and Schlenk techniques. Solution of 0.244 g (0.447 mmol) (^i^Pr_2_N)_2_P—P(SiMe_3_)Li ^.^ 2.6THF in 2 ml DME was added dropwise into solution of 0.059 g (0.202 mmol) [Cp_2_ZrCl_2_] in 2 ml of DME at 233 K. The mixture immediately turned red. The resultant solution was studied using ^31^P-NMR. Then the volume was reduced to about 2 ml and the concentrate stored for 3 days at 269 K. After this time the solution yielded small colourless crystals of (I).

### Refinement

All H atoms were placed in calculated positions and refined as riding on their carrier atoms with respective *U*_iso_(H) values: C—H = 0.98 Å (CH_3_) and *U*_iso_(H) = 1.5 *U*_eq_(C), C—H = 1.00 Å (CH) and *U*_ĩso_(H) = 1.2 *U*_eq_(C). The whole molecule was refined as disordered over two positions with site occupation factors of 0.6447 (8) / 0.3553 (8). Additionally, P–N bond lengths were restrained to be the same and all N atoms displacement ellipsoids were restrained to be equal to improve numerical stability. This kind of disorder was noted already for the structurally similar {P[N(SiMe_3_)_2_](N^i^Pr_2_)_2_ (Bezombes *et al.*, 2004).

### Crystal data

**Table:**

C_24_H_56_N_4_P_2_	*F*(000) = 1032
*M**_r_* = 462.67	*D*_x_ = 1.066 Mg m^−^^3^
Monoclinic, *P*2_1_/*n*	Mo *K*α radiation, λ = 0.71073 Å
Hall symbol: -P 2yn	Cell parameters from 18348 reflections
*a* = 11.601 (2) Å	θ = 3.9–51.8°
*b* = 14.493 (3) Å	µ = 0.17 mm^−^^1^
*c* = 17.280 (4) Å	*T* = 150 K
β = 97.22 (3)°	Block, colourless
*V* = 2882.2 (10) Å^3^	0.38 × 0.23 × 0.21 mm
*Z* = 4	

### Data collection

**Table:**

Stoe IPDS 2 diffractometer	4529 reflections with *I* > 2σ(*I*)
graphite	*R*_int_ = 0.032
Detector resolution: 6.67 pixels mm^-1^	θ_max_ = 26.0°, θ_min_ = 1.8°
rotation scans	*h* = −14→14
20113 measured reflections	*k* = −17→16
5601 independent reflections	*l* = −21→21

### Refinement

**Table:**

Refinement on *F*^2^	Primary atom site location: structure-invariant direct methods
Least-squares matrix: full	Secondary atom site location: difference Fourier map
*R*[*F*^2^ > 2σ(*F*^2^)] = 0.038	Hydrogen site location: inferred from neighbouring sites
*wR*(*F*^2^) = 0.105	H-atom parameters constrained
*S* = 1.03	*w* = 1/[σ^2^(*F*_o_^2^) + (0.0687*P*)^2^ + 0.2046*P*] where *P* = (*F*_o_^2^ + 2*F*_c_^2^)/3
5601 reflections	(Δ/σ)_max_ = 0.001
518 parameters	Δρ_max_ = 0.30 e Å^−^^3^
6 restraints	Δρ_min_ = −0.16 e Å^−^^3^

### Special details

**Table:**

Experimental. ^31^P{^1^H} NMR (162 MHz, external standard 85% H_3_PO_4_) of (I) (THF, C_6_D_6_): 83 p.p.m. (*s*)
Geometry. All e.s.d.'s (except the e.s.d. in the dihedral angle between two l.s. planes) are estimated using the full covariance matrix. The cell e.s.d.'s are taken into account individually in the estimation of e.s.d.'s in distances, angles and torsion angles; correlations between e.s.d.'s in cell parameters are only used when they are defined by crystal symmetry. An approximate (isotropic) treatment of cell e.s.d.'s is used for estimating e.s.d.'s involving l.s. planes.
Refinement. Refinement of *F*^2^ against ALL reflections. The weighted *R*-factor *wR* and goodness of fit *S* are based on *F*^2^, conventional *R*-factors *R* are based on *F*, with *F* set to zero for negative *F*^2^. The threshold expression of *F*^2^ > σ(*F*^2^) is used only for calculating *R*-factors(gt) *etc*. and is not relevant to the choice of reflections for refinement. *R*-factors based on *F*^2^ are statistically about twice as large as those based on *F*, and *R*- factors based on ALL data will be even larger.

### Fractional atomic coordinates and isotropic or equivalent isotropic displacement parameters (Å^2^)

**Table:**

	*x*	*y*	*z*	*U*_iso_*/*U*_eq_	Occ. (<1)
N1	0.2977 (8)	0.7824 (6)	0.1952 (4)	0.0238 (8)	0.6447 (8)
N2	0.4712 (13)	0.7701 (7)	0.3244 (7)	0.0260 (10)	0.6447 (8)
N3	0.6486 (10)	0.8478 (11)	0.1705 (6)	0.0279 (12)	0.6447 (8)
N4	0.5268 (16)	0.6946 (7)	0.0985 (5)	0.0255 (11)	0.6447 (8)
P1	0.42982 (4)	0.81988 (3)	0.23709 (3)	0.02262 (14)	0.6447 (8)
P2	0.57580 (4)	0.74749 (3)	0.18190 (3)	0.02261 (14)	0.6447 (8)
C1	0.2484 (2)	0.68862 (15)	0.21064 (12)	0.0281 (4)	0.6447 (8)
H1A	0.3148	0.6491	0.2335	0.034*	0.6447 (8)
C2	0.1612 (8)	0.6905 (7)	0.2688 (6)	0.0435 (17)	0.6447 (8)
H2A	0.1949	0.7229	0.3161	0.065*	0.6447 (8)
H2B	0.1416	0.6272	0.2821	0.065*	0.6447 (8)
H2C	0.0908	0.7227	0.246	0.065*	0.6447 (8)
C3	0.1894 (7)	0.6384 (7)	0.1372 (4)	0.0425 (15)	0.6447 (8)
H3A	0.2442	0.634	0.0985	0.064*	0.6447 (8)
H3B	0.1206	0.6732	0.115	0.064*	0.6447 (8)
H3C	0.1661	0.5763	0.1514	0.064*	0.6447 (8)
C4	0.2107 (2)	0.85070 (18)	0.16444 (15)	0.0321 (5)	0.6447 (8)
H4A	0.1407	0.8159	0.1406	0.039*	0.6447 (8)
C5	0.2529 (4)	0.9089 (3)	0.0997 (3)	0.0398 (9)	0.6447 (8)
H5A	0.2983	0.8702	0.0683	0.06*	0.6447 (8)
H5B	0.3017	0.9594	0.123	0.06*	0.6447 (8)
H5C	0.1859	0.9345	0.0665	0.06*	0.6447 (8)
C6	0.1714 (8)	0.9144 (6)	0.2269 (6)	0.049 (2)	0.6447 (8)
H6A	0.1154	0.9594	0.2022	0.074*	0.6447 (8)
H6B	0.2389	0.947	0.2538	0.074*	0.6447 (8)
H6C	0.1348	0.8776	0.2647	0.074*	0.6447 (8)
C7	0.4946 (2)	0.67694 (16)	0.34661 (14)	0.0318 (5)	0.6447 (8)
H7A	0.4828	0.6398	0.2975	0.038*	0.6447 (8)
C8	0.4196 (10)	0.6315 (7)	0.4033 (5)	0.0458 (14)	0.6447 (8)
H8A	0.3382	0.6496	0.3894	0.069*	0.6447 (8)
H8B	0.4465	0.6516	0.4566	0.069*	0.6447 (8)
H8C	0.4262	0.5642	0.3999	0.069*	0.6447 (8)
C9	0.6208 (6)	0.6637 (5)	0.3806 (3)	0.0478 (14)	0.6447 (8)
H9A	0.6714	0.6926	0.3463	0.072*	0.6447 (8)
H9B	0.6382	0.5976	0.3851	0.072*	0.6447 (8)
H9C	0.6342	0.6923	0.4324	0.072*	0.6447 (8)
C10	0.4729 (2)	0.83599 (18)	0.39749 (15)	0.0366 (5)	0.6447 (8)
H10A	0.5028	0.7982	0.4441	0.044*	0.6447 (8)
C11	0.3567 (11)	0.8734 (7)	0.4129 (5)	0.059 (3)	0.6447 (8)
H11A	0.3016	0.8223	0.4142	0.088*	0.6447 (8)
H11B	0.3276	0.9165	0.3713	0.088*	0.6447 (8)
H11C	0.3653	0.9056	0.4632	0.088*	0.6447 (8)
C12	0.5594 (10)	0.9140 (8)	0.3935 (6)	0.049 (3)	0.6447 (8)
H12A	0.5581	0.9545	0.4387	0.073*	0.6447 (8)
H12B	0.5383	0.9494	0.3455	0.073*	0.6447 (8)
H12C	0.6375	0.8883	0.3936	0.073*	0.6447 (8)
C13	0.7796 (2)	0.8453 (2)	0.18204 (14)	0.0358 (5)	0.6447 (8)
H13A	0.806	0.9106	0.1779	0.043*	0.6447 (8)
C14	0.8195 (6)	0.8155 (8)	0.2632 (6)	0.050 (2)	0.6447 (8)
H14A	0.9046	0.8159	0.272	0.075*	0.6447 (8)
H14B	0.791	0.7531	0.2713	0.075*	0.6447 (8)
H14C	0.7891	0.858	0.2998	0.075*	0.6447 (8)
C15	0.8412 (9)	0.7908 (7)	0.1267 (4)	0.055 (2)	0.6447 (8)
H15A	0.8153	0.811	0.0732	0.083*	0.6447 (8)
H15B	0.8235	0.7251	0.1319	0.083*	0.6447 (8)
H15C	0.9251	0.8005	0.1385	0.083*	0.6447 (8)
C16	0.6009 (3)	0.93370 (19)	0.13371 (16)	0.0316 (6)	0.6447 (8)
H16A	0.515	0.9244	0.1222	0.038*	0.6447 (8)
C17	0.6190 (3)	1.0128 (3)	0.1930 (2)	0.0440 (8)	0.6447 (8)
H17A	0.5939	0.993	0.2424	0.066*	0.6447 (8)
H17B	0.5732	1.0664	0.1729	0.066*	0.6447 (8)
H17C	0.7015	1.0294	0.2015	0.066*	0.6447 (8)
C18	0.6440 (6)	0.9630 (6)	0.0552 (5)	0.048 (2)	0.6447 (8)
H18A	0.6304	0.9126	0.0174	0.073*	0.6447 (8)
H18B	0.7273	0.9769	0.0643	0.073*	0.6447 (8)
H18C	0.6014	1.018	0.0347	0.073*	0.6447 (8)
C19	0.48180 (18)	0.74866 (17)	0.02342 (11)	0.0287 (4)	0.6447 (8)
H19A	0.4758	0.8148	0.0388	0.034*	0.6447 (8)
C20	0.5698 (9)	0.7446 (6)	−0.0384 (4)	0.053 (2)	0.6447 (8)
H20A	0.6471	0.7631	−0.0138	0.079*	0.6447 (8)
H20B	0.5442	0.7867	−0.0815	0.079*	0.6447 (8)
H20C	0.5732	0.6815	−0.0584	0.079*	0.6447 (8)
C21	0.3607 (8)	0.7197 (4)	−0.0162 (5)	0.0397 (12)	0.6447 (8)
H21A	0.305	0.7219	0.0219	0.059*	0.6447 (8)
H21B	0.3645	0.6567	−0.0364	0.059*	0.6447 (8)
H21C	0.3358	0.762	−0.0593	0.059*	0.6447 (8)
C22	0.5456 (2)	0.59768 (15)	0.08539 (16)	0.0317 (5)	0.6447 (8)
H22A	0.5089	0.5808	0.0318	0.038*	0.6447 (8)
C23	0.6843 (6)	0.5780 (5)	0.0923 (4)	0.061 (2)	0.6447 (8)
H23A	0.7261	0.6368	0.0921	0.091*	0.6447 (8)
H23B	0.7012	0.5404	0.048	0.091*	0.6447 (8)
H23C	0.7094	0.5451	0.1411	0.091*	0.6447 (8)
C24	0.4973 (9)	0.5405 (8)	0.1416 (6)	0.047 (2)	0.6447 (8)
H24A	0.415	0.5556	0.1417	0.071*	0.6447 (8)
H24B	0.5393	0.5516	0.1936	0.071*	0.6447 (8)
H24C	0.5049	0.4754	0.1277	0.071*	0.6447 (8)
N1A	0.2873 (15)	0.7756 (13)	0.2098 (9)	0.0238 (8)	0.3553 (8)
N2A	0.467 (3)	0.7837 (15)	0.3328 (14)	0.0260 (10)	0.3553 (8)
N3A	0.6421 (19)	0.843 (2)	0.1580 (13)	0.0279 (12)	0.3553 (8)
N4A	0.522 (3)	0.7000 (13)	0.0864 (10)	0.0255 (11)	0.3553 (8)
P1A	0.42218 (7)	0.73311 (6)	0.23673 (5)	0.0219 (2)	0.3553 (8)
P2A	0.50767 (7)	0.79679 (6)	0.13605 (5)	0.0230 (2)	0.3553 (8)
C1A	0.1834 (4)	0.7206 (3)	0.2072 (3)	0.0344 (9)	0.3553 (8)
H1B	0.1161	0.7638	0.1963	0.041*	0.3553 (8)
C2A	0.1744 (14)	0.6767 (14)	0.2854 (12)	0.060 (4)	0.3553 (8)
H2D	0.1848	0.724	0.3262	0.09*	0.3553 (8)
H2E	0.2348	0.6295	0.296	0.09*	0.3553 (8)
H2F	0.0977	0.6482	0.2848	0.09*	0.3553 (8)
C3A	0.1765 (13)	0.6541 (12)	0.1395 (10)	0.050 (4)	0.3553 (8)
H3D	0.1806	0.6885	0.0911	0.075*	0.3553 (8)
H3E	0.1029	0.6202	0.1357	0.075*	0.3553 (8)
H3F	0.2414	0.6105	0.1478	0.075*	0.3553 (8)
C4A	0.2637 (4)	0.8765 (3)	0.1849 (3)	0.0301 (9)	0.3553 (8)
H4B	0.3405	0.9087	0.19	0.036*	0.3553 (8)
C5A	0.2117 (6)	0.8852 (6)	0.1017 (5)	0.0424 (16)	0.3553 (8)
H5D	0.2443	0.8375	0.0705	0.064*	0.3553 (8)
H5E	0.2296	0.9463	0.082	0.064*	0.3553 (8)
H5F	0.1273	0.8774	0.0979	0.064*	0.3553 (8)
C6A	0.1864 (13)	0.9294 (11)	0.2368 (11)	0.040 (3)	0.3553 (8)
H6D	0.1932	0.9008	0.2885	0.061*	0.3553 (8)
H6E	0.1053	0.927	0.2128	0.061*	0.3553 (8)
H6F	0.2118	0.9939	0.2418	0.061*	0.3553 (8)
C7A	0.5286 (4)	0.7087 (3)	0.3852 (3)	0.0345 (9)	0.3553 (8)
H7B	0.5669	0.742	0.4323	0.041*	0.3553 (8)
C8A	0.4313 (18)	0.6493 (12)	0.4147 (11)	0.062 (5)	0.3553 (8)
H8D	0.4664	0.6039	0.4526	0.093*	0.3553 (8)
H8E	0.3875	0.6173	0.3705	0.093*	0.3553 (8)
H8F	0.3788	0.6894	0.4396	0.093*	0.3553 (8)
C9A	0.6228 (12)	0.6495 (9)	0.3576 (5)	0.046 (2)	0.3553 (8)
H9D	0.6547	0.6081	0.3997	0.069*	0.3553 (8)
H9E	0.6849	0.6892	0.3428	0.069*	0.3553 (8)
H9F	0.5899	0.613	0.3124	0.069*	0.3553 (8)
C10A	0.4613 (3)	0.8703 (3)	0.3545 (3)	0.0276 (8)	0.3553 (8)
H10B	0.4226	0.906	0.309	0.033*	0.3553 (8)
C11A	0.3793 (19)	0.8709 (11)	0.4182 (9)	0.040 (3)	0.3553 (8)
H11D	0.3753	0.9334	0.4393	0.059*	0.3553 (8)
H11E	0.4089	0.8284	0.4601	0.059*	0.3553 (8)
H11F	0.3014	0.8513	0.3955	0.059*	0.3553 (8)
C12A	0.5729 (16)	0.9193 (11)	0.3839 (10)	0.038 (3)	0.3553 (8)
H12D	0.6325	0.9037	0.3505	0.057*	0.3553 (8)
H12E	0.599	0.8999	0.4375	0.057*	0.3553 (8)
H12F	0.5597	0.9861	0.3826	0.057*	0.3553 (8)
C13A	0.7460 (4)	0.7966 (4)	0.1930 (2)	0.0372 (9)	0.3553 (8)
H13B	0.7228	0.731	0.2	0.045*	0.3553 (8)
C14A	0.8154 (13)	0.8268 (14)	0.2733 (12)	0.052 (4)	0.3553 (8)
H14D	0.7664	0.8185	0.315	0.078*	0.3553 (8)
H14E	0.8376	0.8918	0.2706	0.078*	0.3553 (8)
H14F	0.8855	0.7888	0.2842	0.078*	0.3553 (8)
C15A	0.8263 (18)	0.7953 (13)	0.1260 (10)	0.062 (4)	0.3553 (8)
H15D	0.7832	0.7695	0.0785	0.094*	0.3553 (8)
H15E	0.8949	0.7572	0.1421	0.094*	0.3553 (8)
H15F	0.8507	0.8584	0.1158	0.094*	0.3553 (8)
C16A	0.6525 (5)	0.9454 (4)	0.1463 (3)	0.0403 (13)	0.3553 (8)
H16B	0.7315	0.964	0.1713	0.048*	0.3553 (8)
C17A	0.5667 (6)	1.0084 (5)	0.1782 (6)	0.0556 (19)	0.3553 (8)
H17D	0.5704	0.9992	0.2346	0.083*	0.3553 (8)
H17E	0.4882	0.9943	0.1531	0.083*	0.3553 (8)
H17F	0.5857	1.0727	0.1675	0.083*	0.3553 (8)
C18A	0.6494 (12)	0.9617 (8)	0.0615 (8)	0.043 (4)	0.3553 (8)
H18D	0.7025	0.9187	0.0402	0.064*	0.3553 (8)
H18E	0.6735	1.0252	0.0526	0.064*	0.3553 (8)
H18F	0.5702	0.9519	0.0357	0.064*	0.3553 (8)
C19A	0.4963 (4)	0.6847 (4)	0.0071 (2)	0.0381 (10)	0.3553 (8)
H19B	0.5201	0.6204	−0.0046	0.046*	0.3553 (8)
C20A	0.5551 (16)	0.7475 (9)	−0.0386 (8)	0.048 (4)	0.3553 (8)
H20D	0.6391	0.7435	−0.0226	0.073*	0.3553 (8)
H20E	0.5286	0.8106	−0.0304	0.073*	0.3553 (8)
H20F	0.538	0.7314	−0.0939	0.073*	0.3553 (8)
C21A	0.3689 (15)	0.6925 (9)	−0.0127 (8)	0.054 (3)	0.3553 (8)
H21D	0.3306	0.6468	0.0173	0.081*	0.3553 (8)
H21E	0.3478	0.6812	−0.0686	0.081*	0.3553 (8)
H21F	0.3439	0.7546	0.0002	0.081*	0.3553 (8)
C22A	0.5765 (4)	0.6081 (3)	0.1319 (3)	0.0368 (9)	0.3553 (8)
H22B	0.6075	0.625	0.1867	0.044*	0.3553 (8)
C23A	0.6722 (10)	0.5668 (9)	0.0927 (5)	0.039 (2)	0.3553 (8)
H23D	0.6978	0.6114	0.0558	0.059*	0.3553 (8)
H23E	0.6438	0.5109	0.0646	0.059*	0.3553 (8)
H23F	0.7376	0.551	0.132	0.059*	0.3553 (8)
C24A	0.4815 (16)	0.5278 (14)	0.1335 (8)	0.037 (3)	0.3553 (8)
H24D	0.5187	0.4726	0.1582	0.056*	0.3553 (8)
H24E	0.4479	0.5131	0.0801	0.056*	0.3553 (8)
H24F	0.4199	0.5486	0.1634	0.056*	0.3553 (8)

### Atomic displacement parameters (Å^2^)

**Table:**

	*U*^11^	*U*^22^	*U*^33^	*U*^12^	*U*^13^	*U*^23^
N1	0.0217 (17)	0.0209 (15)	0.030 (3)	−0.0036 (11)	0.0062 (16)	0.0064 (17)
N2	0.0463 (13)	0.015 (3)	0.016 (3)	0.001 (2)	0.0028 (16)	−0.0104 (16)
N3	0.0247 (14)	0.0365 (16)	0.021 (4)	−0.0063 (7)	−0.0041 (19)	0.001 (2)
N4	0.0359 (15)	0.0289 (12)	0.010 (3)	0.0046 (11)	−0.003 (3)	0.0028 (18)
P1	0.0260 (3)	0.0206 (3)	0.0218 (2)	−0.00339 (18)	0.00516 (18)	−0.00181 (18)
P2	0.0209 (2)	0.0270 (3)	0.0196 (2)	−0.00019 (18)	0.00114 (17)	0.00094 (19)
C1	0.0245 (11)	0.0260 (10)	0.0343 (11)	−0.0034 (9)	0.0053 (9)	−0.0004 (8)
C2	0.041 (2)	0.0459 (19)	0.045 (5)	−0.0078 (14)	0.011 (2)	0.004 (2)
C3	0.048 (3)	0.031 (2)	0.043 (3)	−0.0070 (19)	−0.014 (2)	−0.0052 (19)
C4	0.0259 (12)	0.0292 (12)	0.0418 (14)	0.0054 (10)	0.0068 (10)	0.0003 (11)
C5	0.047 (2)	0.036 (2)	0.0387 (15)	0.0144 (14)	0.0125 (18)	0.0133 (14)
C6	0.066 (4)	0.033 (3)	0.053 (4)	0.022 (2)	0.025 (3)	0.010 (3)
C7	0.0427 (13)	0.0284 (12)	0.0230 (11)	−0.0054 (9)	−0.0011 (9)	0.0012 (9)
C8	0.065 (2)	0.038 (3)	0.0368 (19)	−0.009 (2)	0.0171 (18)	0.0101 (19)
C9	0.046 (2)	0.060 (3)	0.034 (3)	−0.005 (2)	−0.005 (2)	0.014 (2)
C10	0.0535 (15)	0.0348 (13)	0.0228 (11)	−0.0129 (11)	0.0095 (10)	−0.0068 (10)
C11	0.059 (5)	0.072 (4)	0.052 (4)	−0.018 (3)	0.034 (4)	−0.035 (3)
C12	0.052 (4)	0.055 (4)	0.042 (3)	−0.016 (2)	0.017 (2)	−0.019 (2)
C13	0.0208 (11)	0.0495 (15)	0.0362 (12)	−0.0068 (11)	0.0004 (9)	0.0027 (11)
C14	0.026 (2)	0.086 (5)	0.038 (3)	−0.016 (2)	−0.0001 (18)	−0.001 (3)
C15	0.021 (3)	0.107 (5)	0.038 (3)	−0.001 (3)	0.0052 (18)	−0.013 (3)
C16	0.0304 (15)	0.0325 (13)	0.0316 (13)	−0.0086 (12)	0.0023 (12)	0.0055 (10)
C17	0.053 (2)	0.0352 (15)	0.0456 (17)	−0.0131 (18)	0.0123 (18)	0.0014 (12)
C18	0.041 (3)	0.065 (5)	0.043 (3)	−0.007 (3)	0.015 (3)	0.026 (3)
C19	0.0316 (10)	0.0341 (12)	0.0204 (9)	0.0028 (9)	0.0028 (8)	−0.0007 (9)
C20	0.050 (3)	0.088 (5)	0.024 (3)	0.005 (2)	0.017 (2)	0.011 (2)
C21	0.037 (2)	0.045 (2)	0.033 (2)	0.0051 (19)	−0.0103 (15)	−0.0037 (15)
C22	0.0362 (12)	0.0302 (11)	0.0292 (12)	0.0067 (9)	0.0060 (10)	−0.0051 (10)
C23	0.054 (3)	0.033 (2)	0.103 (5)	0.027 (2)	0.039 (3)	0.009 (2)
C24	0.049 (3)	0.020 (3)	0.075 (4)	0.003 (3)	0.011 (2)	−0.005 (2)
N1A	0.0217 (17)	0.0209 (15)	0.030 (3)	−0.0036 (11)	0.0062 (16)	0.0064 (17)
N2A	0.0463 (13)	0.015 (3)	0.016 (3)	0.001 (2)	0.0028 (16)	−0.0104 (16)
N3A	0.0247 (14)	0.0365 (16)	0.021 (4)	−0.0063 (7)	−0.0041 (19)	0.001 (2)
N4A	0.0359 (15)	0.0289 (12)	0.010 (3)	0.0046 (11)	−0.003 (3)	0.0028 (18)
P1A	0.0230 (4)	0.0213 (5)	0.0216 (4)	0.0007 (3)	0.0034 (3)	−0.0019 (3)
P2A	0.0206 (4)	0.0281 (5)	0.0202 (4)	0.0022 (3)	0.0020 (3)	0.0014 (3)
C1A	0.023 (2)	0.035 (2)	0.047 (3)	−0.0046 (18)	0.0092 (17)	−0.0086 (19)
C2A	0.041 (6)	0.103 (11)	0.041 (7)	−0.033 (6)	0.024 (5)	−0.003 (6)
C3A	0.046 (5)	0.037 (6)	0.070 (6)	−0.021 (5)	0.023 (4)	−0.018 (4)
C4A	0.027 (2)	0.028 (2)	0.035 (2)	0.0026 (17)	0.0018 (18)	−0.0026 (17)
C5A	0.047 (4)	0.046 (4)	0.034 (3)	0.014 (3)	0.005 (3)	0.004 (3)
C6A	0.033 (3)	0.042 (5)	0.048 (4)	0.011 (3)	0.011 (3)	−0.017 (4)
C7A	0.042 (2)	0.034 (2)	0.027 (2)	0.0029 (19)	0.0016 (18)	0.0029 (18)
C8A	0.079 (8)	0.043 (7)	0.066 (9)	0.008 (6)	0.014 (5)	0.030 (6)
C9A	0.061 (4)	0.047 (4)	0.028 (5)	0.029 (3)	−0.003 (4)	0.010 (3)
C10A	0.0306 (19)	0.0257 (19)	0.026 (2)	−0.0025 (15)	0.0035 (15)	−0.0066 (17)
C11A	0.047 (7)	0.036 (4)	0.035 (4)	−0.002 (3)	0.001 (4)	−0.007 (3)
C12A	0.040 (5)	0.031 (4)	0.038 (5)	−0.014 (4)	−0.014 (5)	−0.011 (4)
C13A	0.026 (2)	0.053 (3)	0.032 (2)	0.001 (2)	0.0030 (17)	0.001 (2)
C14A	0.056 (6)	0.056 (5)	0.035 (5)	0.000 (4)	−0.027 (4)	−0.003 (4)
C15A	0.017 (5)	0.077 (7)	0.091 (9)	0.008 (4)	0.002 (4)	0.018 (6)
C16A	0.031 (3)	0.044 (3)	0.044 (3)	−0.012 (3)	0.000 (3)	0.008 (2)
C17A	0.063 (5)	0.027 (3)	0.080 (5)	−0.009 (4)	0.022 (4)	0.009 (3)
C18A	0.056 (7)	0.029 (6)	0.037 (5)	−0.017 (4)	−0.016 (4)	−0.001 (4)
C19A	0.040 (2)	0.053 (3)	0.0205 (18)	0.007 (2)	0.0033 (16)	−0.0064 (19)
C20A	0.057 (7)	0.047 (6)	0.040 (7)	−0.014 (4)	0.002 (4)	−0.017 (4)
C21A	0.040 (4)	0.100 (10)	0.021 (3)	0.022 (6)	−0.002 (2)	−0.001 (5)
C22A	0.042 (2)	0.039 (2)	0.030 (2)	0.0147 (19)	0.0054 (19)	−0.0013 (19)
C23A	0.035 (4)	0.053 (5)	0.032 (4)	−0.012 (3)	0.011 (3)	−0.009 (3)
C24A	0.048 (5)	0.027 (6)	0.038 (4)	−0.010 (3)	0.013 (3)	0.000 (3)

### Geometric parameters (Å, °)

**Table:**

N1—C4	1.465 (10)	N1A—C1A	1.441 (17)
N1—C1	1.512 (8)	N1A—C4A	1.539 (18)
N1—P1	1.699 (9)	N1A—P1A	1.692 (19)
N2—C7	1.420 (12)	N2A—C10A	1.313 (19)
N2—C10	1.582 (7)	N2A—C7A	1.53 (3)
N2—P1	1.687 (14)	N2A—P1A	1.83 (2)
N3—C16	1.474 (17)	N3A—C13A	1.45 (3)
N3—C13	1.508 (12)	N3A—C16A	1.50 (3)
N3—P2	1.705 (11)	N3A—P2A	1.697 (14)
N4—C22	1.444 (9)	N4A—C19A	1.383 (17)
N4—C19	1.549 (10)	N4A—C22A	1.63 (2)
N4—P2	1.667 (8)	N4A—P2A	1.664 (14)
P1—P2	2.2988 (8)	P1A—P2A	2.3013 (13)
C1—C2	1.513 (10)	C1A—C2A	1.509 (18)
C1—C3	1.546 (8)	C1A—C3A	1.510 (18)
C1—H1A	1	C1A—H1B	1
C2—H2A	0.98	C2A—H2D	0.98
C2—H2B	0.98	C2A—H2E	0.98
C2—H2C	0.98	C2A—H2F	0.98
C3—H3A	0.98	C3A—H3D	0.98
C3—H3B	0.98	C3A—H3E	0.98
C3—H3C	0.98	C3A—H3F	0.98
C4—C5	1.530 (4)	C4A—C5A	1.494 (10)
C4—C6	1.533 (10)	C4A—C6A	1.548 (17)
C4—H4A	1	C4A—H4B	1
C5—H5A	0.98	C5A—H5D	0.98
C5—H5B	0.98	C5A—H5E	0.98
C5—H5C	0.98	C5A—H5F	0.98
C6—H6A	0.98	C6A—H6D	0.98
C6—H6B	0.98	C6A—H6E	0.98
C6—H6C	0.98	C6A—H6F	0.98
C7—C9	1.520 (7)	C7A—C9A	1.513 (12)
C7—C8	1.537 (10)	C7A—C8A	1.56 (2)
C7—H7A	1	C7A—H7B	1
C8—H8A	0.98	C8A—H8D	0.98
C8—H8B	0.98	C8A—H8E	0.98
C8—H8C	0.98	C8A—H8F	0.98
C9—H9A	0.98	C9A—H9D	0.98
C9—H9B	0.98	C9A—H9E	0.98
C9—H9C	0.98	C9A—H9F	0.98
C10—C11	1.508 (12)	C10A—C12A	1.507 (17)
C10—C12	1.518 (12)	C10A—C11A	1.54 (2)
C10—H10A	1	C10A—H10B	1
C11—H11A	0.98	C11A—H11D	0.98
C11—H11B	0.98	C11A—H11E	0.98
C11—H11C	0.98	C11A—H11F	0.98
C12—H12A	0.98	C12A—H12D	0.98
C12—H12B	0.98	C12A—H12E	0.98
C12—H12C	0.98	C12A—H12F	0.98
C13—C14	1.484 (10)	C13A—C15A	1.574 (18)
C13—C15	1.490 (9)	C13A—C14A	1.58 (2)
C13—H13A	1	C13A—H13B	1
C14—H14A	0.98	C14A—H14D	0.98
C14—H14B	0.98	C14A—H14E	0.98
C14—H14C	0.98	C14A—H14F	0.98
C15—H15A	0.98	C15A—H15D	0.98
C15—H15B	0.98	C15A—H15E	0.98
C15—H15C	0.98	C15A—H15F	0.98
C16—C17	1.534 (5)	C16A—C18A	1.479 (15)
C16—C18	1.562 (7)	C16A—C17A	1.505 (11)
C16—H16A	1	C16A—H16B	1
C17—H17A	0.98	C17A—H17D	0.98
C17—H17B	0.98	C17A—H17E	0.98
C17—H17C	0.98	C17A—H17F	0.98
C18—H18A	0.98	C18A—H18D	0.98
C18—H18B	0.98	C18A—H18E	0.98
C18—H18C	0.98	C18A—H18F	0.98
C19—C21	1.541 (9)	C19A—C20A	1.432 (15)
C19—C20	1.569 (9)	C19A—C21A	1.478 (17)
C19—H19A	1	C19A—H19B	1
C20—H20A	0.98	C20A—H20D	0.98
C20—H20B	0.98	C20A—H20E	0.98
C20—H20C	0.98	C20A—H20F	0.98
C21—H21A	0.98	C21A—H21D	0.98
C21—H21B	0.98	C21A—H21E	0.98
C21—H21C	0.98	C21A—H21F	0.98
C22—C24	1.442 (10)	C22A—C23A	1.496 (12)
C22—C23	1.624 (7)	C22A—C24A	1.61 (2)
C22—H22A	1	C22A—H22B	1
C23—H23A	0.98	C23A—H23D	0.98
C23—H23B	0.98	C23A—H23E	0.98
C23—H23C	0.98	C23A—H23F	0.98
C24—H24A	0.98	C24A—H24D	0.98
C24—H24B	0.98	C24A—H24E	0.98
C24—H24C	0.98	C24A—H24F	0.98
			
C4—N1—C1	114.3 (6)	C1A—N1A—C4A	113.4 (12)
C4—N1—P1	118.8 (6)	C1A—N1A—P1A	123.3 (11)
C1—N1—P1	123.6 (6)	C4A—N1A—P1A	123.3 (10)
C7—N2—C10	112.0 (9)	C10A—N2A—C7A	122.9 (17)
C7—N2—P1	132.2 (6)	C10A—N2A—P1A	128.6 (19)
C10—N2—P1	115.4 (7)	C7A—N2A—P1A	108.0 (10)
C16—N3—C13	113.3 (6)	C13A—N3A—C16A	116.1 (9)
C16—N3—P2	127.2 (7)	C13A—N3A—P2A	127.1 (18)
C13—N3—P2	118.0 (11)	C16A—N3A—P2A	117 (2)
C22—N4—C19	113.7 (5)	C19A—N4A—C22A	111.7 (10)
C22—N4—P2	122.8 (7)	C19A—N4A—P2A	128.5 (15)
C19—N4—P2	122.3 (6)	C22A—N4A—P2A	119.8 (11)
N2—P1—N1	112.6 (5)	N1A—P1A—N2A	104.5 (10)
N2—P1—P2	92.0 (4)	N1A—P1A—P2A	96.7 (4)
N1—P1—P2	110.4 (3)	N2A—P1A—P2A	115.2 (9)
N4—P2—N3	114.3 (6)	N4A—P2A—N3A	107.5 (18)
N4—P2—P1	112.3 (6)	N4A—P2A—P1A	97.6 (10)
N3—P2—P1	93.5 (6)	N3A—P2A—P1A	117.5 (8)
N1—C1—C2	113.6 (5)	N1A—C1A—C2A	110.9 (9)
N1—C1—C3	114.6 (5)	N1A—C1A—C3A	109.9 (9)
C2—C1—C3	106.9 (5)	C2A—C1A—C3A	114.9 (11)
N1—C1—H1A	107.1	N1A—C1A—H1B	106.9
C2—C1—H1A	107.1	C2A—C1A—H1B	106.9
C3—C1—H1A	107.1	C3A—C1A—H1B	106.9
C1—C2—H2A	109.5	C1A—C2A—H2D	109.5
C1—C2—H2B	109.5	C1A—C2A—H2E	109.5
H2A—C2—H2B	109.5	H2D—C2A—H2E	109.5
C1—C2—H2C	109.5	C1A—C2A—H2F	109.5
H2A—C2—H2C	109.5	H2D—C2A—H2F	109.5
H2B—C2—H2C	109.5	H2E—C2A—H2F	109.5
C1—C3—H3A	109.5	C1A—C3A—H3D	109.5
C1—C3—H3B	109.5	C1A—C3A—H3E	109.5
H3A—C3—H3B	109.5	H3D—C3A—H3E	109.5
C1—C3—H3C	109.5	C1A—C3A—H3F	109.5
H3A—C3—H3C	109.5	H3D—C3A—H3F	109.5
H3B—C3—H3C	109.5	H3E—C3A—H3F	109.5
N1—C4—C5	111.6 (3)	C5A—C4A—N1A	112.6 (8)
N1—C4—C6	113.8 (5)	C5A—C4A—C6A	108.8 (8)
C5—C4—C6	109.5 (4)	N1A—C4A—C6A	113.9 (9)
N1—C4—H4A	107.2	C5A—C4A—H4B	107
C5—C4—H4A	107.2	N1A—C4A—H4B	107
C6—C4—H4A	107.2	C6A—C4A—H4B	107
C4—C5—H5A	109.5	C4A—C5A—H5D	109.5
C4—C5—H5B	109.5	C4A—C5A—H5E	109.5
H5A—C5—H5B	109.5	H5D—C5A—H5E	109.5
C4—C5—H5C	109.5	C4A—C5A—H5F	109.5
H5A—C5—H5C	109.5	H5D—C5A—H5F	109.5
H5B—C5—H5C	109.5	H5E—C5A—H5F	109.5
C4—C6—H6A	109.5	C4A—C6A—H6D	109.5
C4—C6—H6B	109.5	C4A—C6A—H6E	109.5
H6A—C6—H6B	109.5	H6D—C6A—H6E	109.5
C4—C6—H6C	109.5	C4A—C6A—H6F	109.5
H6A—C6—H6C	109.5	H6D—C6A—H6F	109.5
H6B—C6—H6C	109.5	H6E—C6A—H6F	109.5
N2—C7—C9	111.5 (7)	C9A—C7A—N2A	121.2 (9)
N2—C7—C8	118.4 (7)	C9A—C7A—C8A	111.9 (9)
C9—C7—C8	107.1 (5)	N2A—C7A—C8A	106.2 (13)
N2—C7—H7A	106.4	C9A—C7A—H7B	105.4
C9—C7—H7A	106.4	N2A—C7A—H7B	105.4
C8—C7—H7A	106.4	C8A—C7A—H7B	105.4
C7—C8—H8A	109.5	C7A—C8A—H8D	109.5
C7—C8—H8B	109.5	C7A—C8A—H8E	109.5
H8A—C8—H8B	109.5	H8D—C8A—H8E	109.5
C7—C8—H8C	109.5	C7A—C8A—H8F	109.5
H8A—C8—H8C	109.5	H8D—C8A—H8F	109.5
H8B—C8—H8C	109.5	H8E—C8A—H8F	109.5
C7—C9—H9A	109.5	C7A—C9A—H9D	109.5
C7—C9—H9B	109.5	C7A—C9A—H9E	109.5
H9A—C9—H9B	109.5	H9D—C9A—H9E	109.5
C7—C9—H9C	109.5	C7A—C9A—H9F	109.5
H9A—C9—H9C	109.5	H9D—C9A—H9F	109.5
H9B—C9—H9C	109.5	H9E—C9A—H9F	109.5
C11—C10—C12	110.6 (7)	N2A—C10A—C12A	118.5 (15)
C11—C10—N2	115.9 (7)	N2A—C10A—C11A	105.4 (14)
C12—C10—N2	110.8 (7)	C12A—C10A—C11A	109.8 (11)
C11—C10—H10A	106.3	N2A—C10A—H10B	107.6
C12—C10—H10A	106.3	C12A—C10A—H10B	107.6
N2—C10—H10A	106.3	C11A—C10A—H10B	107.6
C10—C11—H11A	109.5	C10A—C11A—H11D	109.5
C10—C11—H11B	109.5	C10A—C11A—H11E	109.5
H11A—C11—H11B	109.5	H11D—C11A—H11E	109.5
C10—C11—H11C	109.5	C10A—C11A—H11F	109.5
H11A—C11—H11C	109.5	H11D—C11A—H11F	109.5
H11B—C11—H11C	109.5	H11E—C11A—H11F	109.5
C10—C12—H12A	109.5	C10A—C12A—H12D	109.5
C10—C12—H12B	109.5	C10A—C12A—H12E	109.5
H12A—C12—H12B	109.5	H12D—C12A—H12E	109.5
C10—C12—H12C	109.5	C10A—C12A—H12F	109.5
H12A—C12—H12C	109.5	H12D—C12A—H12F	109.5
H12B—C12—H12C	109.5	H12E—C12A—H12F	109.5
C14—C13—C15	109.6 (6)	N3A—C13A—C15A	103.8 (12)
C14—C13—N3	108.7 (5)	N3A—C13A—C14A	123.0 (13)
C15—C13—N3	119.0 (6)	C15A—C13A—C14A	111.5 (10)
C14—C13—H13A	106.3	N3A—C13A—H13B	105.8
C15—C13—H13A	106.3	C15A—C13A—H13B	105.8
N3—C13—H13A	106.3	C14A—C13A—H13B	105.8
C13—C14—H14A	109.5	C13A—C14A—H14D	109.5
C13—C14—H14B	109.5	C13A—C14A—H14E	109.5
H14A—C14—H14B	109.5	H14D—C14A—H14E	109.5
C13—C14—H14C	109.5	C13A—C14A—H14F	109.5
H14A—C14—H14C	109.5	H14D—C14A—H14F	109.5
H14B—C14—H14C	109.5	H14E—C14A—H14F	109.5
C13—C15—H15A	109.5	C13A—C15A—H15D	109.5
C13—C15—H15B	109.5	C13A—C15A—H15E	109.5
H15A—C15—H15B	109.5	H15D—C15A—H15E	109.5
C13—C15—H15C	109.5	C13A—C15A—H15F	109.5
H15A—C15—H15C	109.5	H15D—C15A—H15F	109.5
H15B—C15—H15C	109.5	H15E—C15A—H15F	109.5
N3—C16—C17	109.3 (5)	C18A—C16A—N3A	107.3 (11)
N3—C16—C18	117.4 (6)	C18A—C16A—C17A	109.3 (8)
C17—C16—C18	110.2 (4)	N3A—C16A—C17A	118.8 (11)
N3—C16—H16A	106.4	C18A—C16A—H16B	106.9
C17—C16—H16A	106.4	N3A—C16A—H16B	106.9
C18—C16—H16A	106.4	C17A—C16A—H16B	106.9
C16—C17—H17A	109.5	C16A—C17A—H17D	109.5
C16—C17—H17B	109.5	C16A—C17A—H17E	109.5
H17A—C17—H17B	109.5	H17D—C17A—H17E	109.5
C16—C17—H17C	109.5	C16A—C17A—H17F	109.5
H17A—C17—H17C	109.5	H17D—C17A—H17F	109.5
H17B—C17—H17C	109.5	H17E—C17A—H17F	109.5
C16—C18—H18A	109.5	C16A—C18A—H18D	109.5
C16—C18—H18B	109.5	C16A—C18A—H18E	109.5
H18A—C18—H18B	109.5	H18D—C18A—H18E	109.5
C16—C18—H18C	109.5	C16A—C18A—H18F	109.5
H18A—C18—H18C	109.5	H18D—C18A—H18F	109.5
H18B—C18—H18C	109.5	H18E—C18A—H18F	109.5
C21—C19—N4	115.3 (7)	N4A—C19A—C20A	112.6 (14)
C21—C19—C20	108.8 (5)	N4A—C19A—C21A	107.6 (16)
N4—C19—C20	111.8 (7)	C20A—C19A—C21A	110.9 (10)
C21—C19—H19A	106.8	N4A—C19A—H19B	108.5
N4—C19—H19A	106.8	C20A—C19A—H19B	108.5
C20—C19—H19A	106.8	C21A—C19A—H19B	108.5
C19—C20—H20A	109.5	C19A—C20A—H20D	109.5
C19—C20—H20B	109.5	C19A—C20A—H20E	109.5
H20A—C20—H20B	109.5	H20D—C20A—H20E	109.5
C19—C20—H20C	109.5	C19A—C20A—H20F	109.5
H20A—C20—H20C	109.5	H20D—C20A—H20F	109.5
H20B—C20—H20C	109.5	H20E—C20A—H20F	109.5
C19—C21—H21A	109.5	C19A—C21A—H21D	109.5
C19—C21—H21B	109.5	C19A—C21A—H21E	109.5
H21A—C21—H21B	109.5	H21D—C21A—H21E	109.5
C19—C21—H21C	109.5	C19A—C21A—H21F	109.5
H21A—C21—H21C	109.5	H21D—C21A—H21F	109.5
H21B—C21—H21C	109.5	H21E—C21A—H21F	109.5
C24—C22—N4	112.0 (7)	C23A—C22A—C24A	105.5 (8)
C24—C22—C23	108.3 (5)	C23A—C22A—N4A	111.8 (12)
N4—C22—C23	109.1 (8)	C24A—C22A—N4A	112.0 (14)
C24—C22—H22A	109.1	C23A—C22A—H22B	109.1
N4—C22—H22A	109.1	C24A—C22A—H22B	109.1
C23—C22—H22A	109.1	N4A—C22A—H22B	109.1
C22—C23—H23A	109.5	C22A—C23A—H23D	109.5
C22—C23—H23B	109.5	C22A—C23A—H23E	109.5
H23A—C23—H23B	109.5	H23D—C23A—H23E	109.5
C22—C23—H23C	109.5	C22A—C23A—H23F	109.5
H23A—C23—H23C	109.5	H23D—C23A—H23F	109.5
H23B—C23—H23C	109.5	H23E—C23A—H23F	109.5
C22—C24—H24A	109.5	C22A—C24A—H24D	109.5
C22—C24—H24B	109.5	C22A—C24A—H24E	109.5
H24A—C24—H24B	109.5	H24D—C24A—H24E	109.5
C22—C24—H24C	109.5	C22A—C24A—H24F	109.5
H24A—C24—H24C	109.5	H24D—C24A—H24F	109.5
H24B—C24—H24C	109.5	H24E—C24A—H24F	109.5
			
N1—P1—P2—N3	132.8 (4)	C1—N1—C4—C6	96.8 (7)
N1—P1—P2—N4	14.7 (5)	P1—N1—C4—C6	−63.7 (5)
N2—P1—P2—N3	−112.2 (6)	P1—N2—C7—C9	116.3 (12)
N2—P1—P2—N4	129.7 (6)	P1—N2—C7—C8	−118.7 (11)
C7—N2—P1—N1	62.6 (14)	C10—N2—C7—C9	−70.7 (10)
C10—N2—P1—N1	−110.2 (9)	C10—N2—C7—C8	54.2 (12)
C7—N2—P1—P2	−50.6 (13)	C7—N2—C10—C11	−109.2 (10)
C10—N2—P1—P2	136.6 (8)	P1—N2—C10—C11	65.0 (11)
C4—N1—P1—N2	131.3 (4)	C7—N2—C10—C12	123.8 (9)
C1—N1—P1—N2	−27.2 (7)	P1—N2—C10—C12	−62.0 (11)
C4—N1—P1—P2	−127.5 (4)	C16—N3—C13—C14	132.0 (8)
C1—N1—P1—P2	74.0 (5)	P2—N3—C13—C14	−60.8 (9)
C22—N4—P2—N3	130.9 (12)	C16—N3—C13—C15	−101.7 (9)
C19—N4—P2—N3	−35.4 (16)	P2—N3—C13—C15	65.5 (9)
C22—N4—P2—P1	−124.2 (12)	C13—N3—C16—C17	−73.1 (9)
C19—N4—P2—P1	69.5 (13)	P2—N3—C16—C17	121.1 (8)
C16—N3—P2—N4	65.3 (14)	C13—N3—C16—C18	53.2 (9)
C13—N3—P2—N4	−99.9 (9)	P2—N3—C16—C18	−112.5 (10)
C16—N3—P2—P1	−51.1 (10)	C22—N4—C19—C21	64.3 (13)
C13—N3—P2—P1	143.7 (7)	P2—N4—C19—C21	−128.3 (11)
C4—N1—C1—C2	−59.5 (7)	C22—N4—C19—C20	−60.7 (13)
P1—N1—C1—C2	99.9 (7)	P2—N4—C19—C20	106.8 (11)
C4—N1—C1—C3	63.8 (7)	C19—N4—C22—C24	−133.6 (10)
P1—N1—C1—C3	−136.8 (6)	P2—N4—C22—C24	59.0 (15)
C1—N1—C4—C5	−138.7 (4)	C19—N4—C22—C23	106.5 (11)
P1—N1—C4—C5	60.8 (5)	P2—N4—C22—C23	−60.9 (13)
